# High-performance near-infrared photodetectors based on gate-controlled graphene–germanium Schottky junction with split active junction

**DOI:** 10.1515/nanoph-2021-0738

**Published:** 2022-01-07

**Authors:** Cihyun Kim, Tae Jin Yoo, Min Gyu Kwon, Kyoung Eun Chang, Hyeon Jun Hwang, Byoung Hun Lee

**Affiliations:** Department of Electrical Engineering, Pohang University of Science and Technology, 77, Cheongam-ro, Nam-gu, Pohang-si, Gyeongsangbuk-do, 37673, Republic of Korea; School of Materials Science and Engineering, Gwangju Institute of Science and Technology, 123, Cheomdangwagi-ro, Buk-gu, Gwangju, 61005, Republic of Korea

**Keywords:** germanium, graphene, photodetector, Schottky junction, split active junction

## Abstract

The structure of a gate-controlled graphene/germanium hybrid photodetector was optimized by splitting the active region to achieve highly sensitive infrared detection capability. The strengthened internal electric field in the split active junctions enabled efficient collection of photocarriers, resulting in a responsivity of 2.02 A W^−1^ and a specific detectivity of 5.28 × 10^10^ Jones with reduced dark current and improved external quantum efficiency; these results are more than doubled compared with the responsivity of 0.85 A W^−1^ and detectivity of 1.69 × 10^10^ Jones for a single active junction device. The responsivity of the optimized structure is 1.7, 2.7, and 39 times higher than that of previously reported graphene/Ge with Al_2_O_3_ interfacial layer, gate-controlled graphene/Ge, and simple graphene/Ge heterostructure photodetectors, respectively.

## 1 Introduction

Various graphene/semiconductor Schottky junction photodetectors have been investigated to combine the benefits of graphene and semiconductors in terms of photonic responses [1], [2], [3], [4], [5], [6], [7], [8], [9], [10], [11], [12]. In particular, graphene/Ge heterojunctions have been used in infrared wavelength regions owing to their simple structure, exceptional optoelectronic properties, and direct compatibility with high-speed integrated circuits [13], [14], [15].

Zeng et al. demonstrated a graphene/Ge Schottky junction-based infrared photodetector with a responsivity of 51.8 mA W^−1^ operating at zero bias voltage [13]. Chang et al. reported a gate-modulated graphene/Ge Schottky junction photodetector with a responsivity of 750 mA W^−1^. In this device, a self-amplification process is used to obtain a high photocurrent with a concurrent reduction in the dark current. The self-amplification mechanism is induced by asymmetric carrier transport and lifetime in the graphene and Ge regions [15]. Currently, the graphene/Ge junction photodetectors have a comparatively smaller gain than graphene/Si junction photodetectors, and the responsivity remains limited due to low external quantum efficiency (EQE) of 60% because of the long transit time of the minority carrier in the depletion region of Ge [12, 15]. These results suggest that a primary problem with a gate-controlled graphene/Ge Schottky junction photodetector is the inefficient transport of photogenerated carriers.

Recently, various approaches to improve carrier transport in graphene/semiconductor junctions through electric field engineering have been reported [16], [17], [18], [19], [20], [21], [22], [23], [24], [25]. Bartolomeo et al. reported that a graphene/silicon-nanotip heterojunction creates a higher local electric field in the Si substrate, which improves the photo-charge separation [17]. Furthermore, several groups have investigated the spatial distribution of photocurrents in graphene/semiconductor heterojunctions to elucidate the mechanism of photocurrent transport using scanning photocurrent microscopy (SPCM). Unsuree et al. reported that the strong band bending of a silicon substrate induced by local strengthening of the electric field at the graphene edge influences the transit of photogenerated carriers in graphene/Si photodiodes [22]. Riazimehr et al. reported that the effective collection of photogenerated carriers has been achieved with reduced surface recombination and an extended lifetime of minority carriers by inducing formation of an inversion layer in the adjacent graphene/SiO_2_/Si structure [19, 21]. Although the interface between graphene and silicon may differ from that between graphene and germanium, these studies provide a suitable design guideline for graphene/Ge junction photodetectors.

In this study, gate-controlled hybrid infrared photodetectors with two different active regions, that is, a single active junction and a split active junction, were investigated. The split active junction structure was implemented to improve the EQE and reduce the dark current. The strengthened internal electric field enables photocarrier extraction with an internal gain, which was investigated using the SPCM and numerical simulation. As a result, a split active region improves the responsivity and specific detectivity to 2.02 A W^−1^ and 5.28 × 10^10^ Jones from 0.85 A W^−1^ and 1.69 × 10^10^ Jones for a single active device. This result is 1.7–39 times higher than the previously-reported responsivity of a graphene/Ge photodetector.

## 2 Experimental details

**Figure 1:**
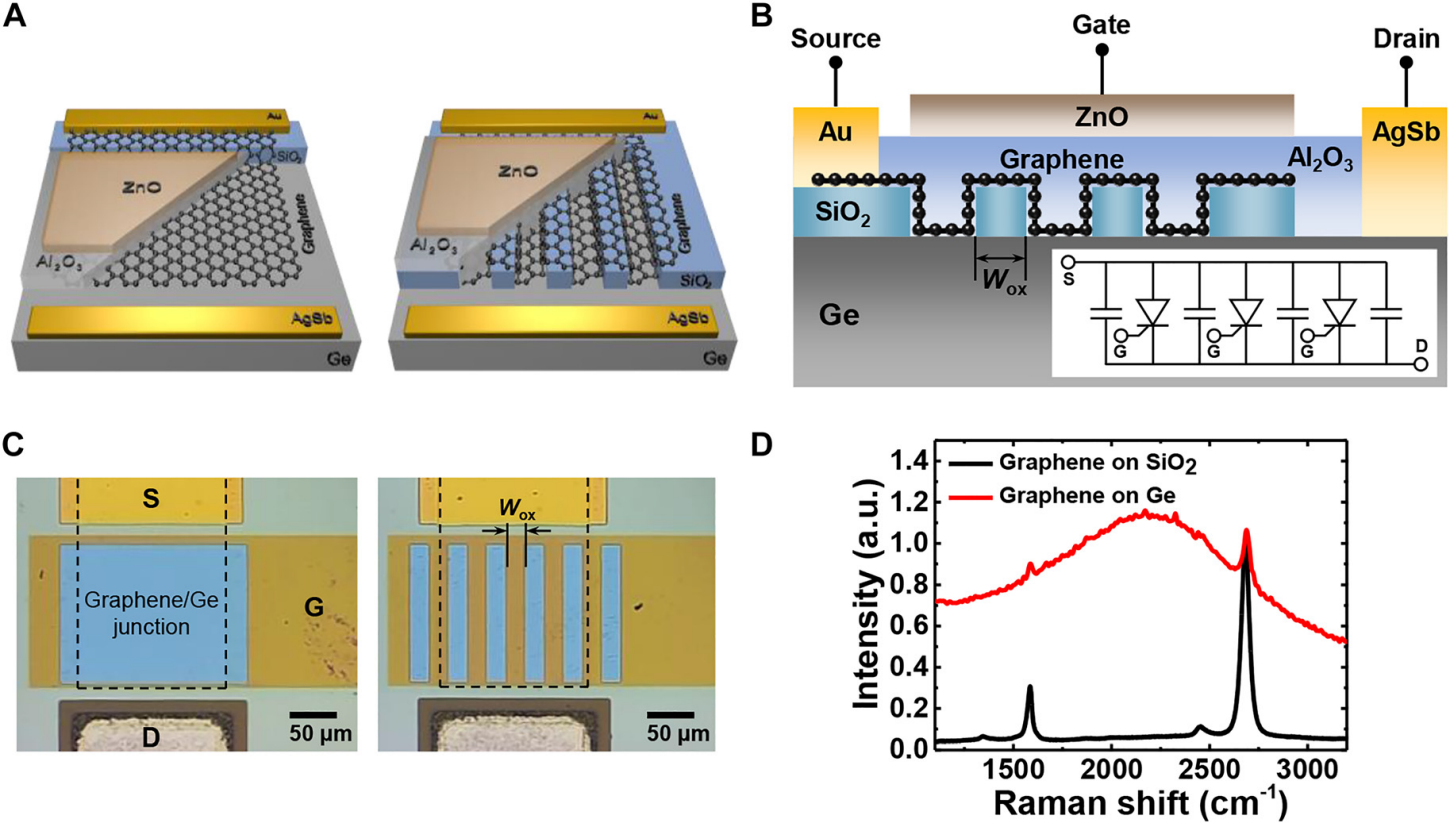
(A) Schematic diagrams of the single (left) and split (right) active junction structure of the gate-controlled graphene/Ge Schottky junction photodetector. (B) Cross-sectional schematic of a split active junction device. *W*_ox_ represents the width of the interdigitated oxide layer, and four devices (*W*_ox_ = 0, 10, 20, or 30 μm) were fabricated. Inset: equivalent circuit, where the capacitors are connected in parallel with the gate-controlled Schottky diodes. (C) Optical images of fabricated single (left) and split (right) active junction devices. Black dashed rectangle: graphene area. (D) Raman spectrum of monolayer graphene on SiO_2_ and Ge substrate.

Figure 1A shows the fabricated devices with two different active regions, where the single active structure comprises only graphene/Ge Schottky junctions, and the split active structure is divided into graphene/Ge regions and graphene/SiO_2_/Ge regions. A cross-sectional schematic of the split active-junction device is shown in Figure 1B. The inset of Figure 1B is the equivalent circuit of the split active junction device comprising gate-controlled graphene/Ge Schottky diodes and graphene/SiO_2_/Ge capacitors separated by an interdigitated insulating layer.

For device fabrication, a 100-nm-thick SiO_2_ layer was deposited on an *n*-type Ge substrate (resistivity 2.5–2.7 Ω cm) using plasma-enhanced chemical vapor deposition (PECVD). The native oxide of the Ge could affect the interface quality; therefore, the substrate was immersed in deionized water at room temperature for 24 h to remove the native oxide; next, a 100-nm layer of SiO_2_ was deposited on it [26, 27]. Thereafter, the SiO_2_ layer was patterned to expose the Ge surface using photolithography and buffered oxide etching (BOE) process. Four types of devices with oxide widths of 0, 10, 20, and 30 μm were fabricated. Optical microscope images were obtained for the fabricated devices with oxide widths of 0 and 20 μm, as shown in Figure 1C.

Immediately afterward, a thermal CVD-grown monolayer graphene sheet was wet-transferred onto the patterned substrate using a polymethylmethacrylate (PMMA) sacrificial layer to minimize the amount of native oxide at the graphene/Ge interface. Following the transfer and acetone rinsing, graphene channel patterns were formed using 30-nm-thick Au as a hard mask and O_2_-plasma etching. Raman spectra were obtained from graphene on SiO_2_ and Ge substrates, as shown in Figure 1D. The quality of the graphene film was confirmed by the presence of a 2D band peak *I*_2D_ at 2680 cm^−1^, G band peak *I*_G_ at 1580 cm^−1^, and D band peak *I*_D_ at 1350 cm^−1^. Furthermore, the 2D and G bands have an intensity ratio *I*_2D_/*I*_G_ > 2, confirming that graphene is a monolayer.

Subsequently, a 100-nm-thick Au source electrode was formed on the graphene channel using an e-beam evaporator and photolithography process. For the drain electrode, a 45-nm-thick AgSb alloy (Ag with 1% Sb) was formed on the Ge region using a thermal evaporator and a lift-off process, followed by rapid thermal annealing (RTA) for 5 min in a N_2_ atmosphere at 450 °C. After the formation of the AgSb drain contact, the contact resistance of the AgSb electrode was measured using the circular transfer length method [28]. The measured specific contact resistivity was *ρ*_c_ = 4.7 × 10^−7^ Ω cm^2^ (refer to Figure S1 in the Supporting Information). This is a relatively low contact resistance for moderately-doped *n*-type Ge, considering that highly doped *n*-Ge (*N*_D_ > 1 × 10^19^ cm^−3^) was used to obtain a low specific contact resistivity of *ρ*_c_ = 1.68 × 10^−7^ Ω cm^2^ by using a NiGe ohmic contact [29].

After forming the electrodes, a 50-nm-thick Al_2_O_3_ layer was deposited at 200 °C using atomic layer deposition (ALD) (Lucida D100, NCD tech) as the gate dielectric. A 50-nm-thick transparent ZnO film was deposited using ALD, and the ZnO top gate was then patterned using photolithography and HCl wet etching. The growth rates of Al_2_O_3_ and ZnO measured using spectroscopic ellipsometry were ∼1 Å and 1.7 Å per cycle, respectively. Finally, passivation annealing was performed at 300 °C under high vacuum conditions for 1 h to remove any remaining water molecules from the interface of graphene and Ge and to densify the Al_2_O_3_ layer.

The current–voltage (*I*–*V*) characteristics of the photodetector were measured using a semiconductor parameter analyzer (Keithley 4200). All characterizations were performed under ambient conditions in air (temperature *T* = 300 K, pressure *P* = 1 atm). The photoresponse characteristics were measured using solid-state laser diodes at wavelengths *λ* = 520, 850, 1060, 1310, 1550, and 1625 nm. For SPCM measurements, a solid-state laser (*λ* = 1550 nm) was focused through the objective lens (Olympus LUCPlanFLN, NA = 0.6, 40×) of a microscope and scanned using a two-axis galvanometer scanner. The photocurrent signals were collected using a picoammeter (Keithley 2502) as a function of the laser position. The diffraction-limited spot size at *λ* = 1550 nm was ∼3.5 μm. The incident light power was monitored using an optical power meter (Newport 1918-C) and a thermopile sensor (Newport 919P-003-10). Figure S2 in the Supporting Information section provides more information on the experimental setup used to characterize the photoresponsivity.

## 3 Results and discussion

**Figure 2:**
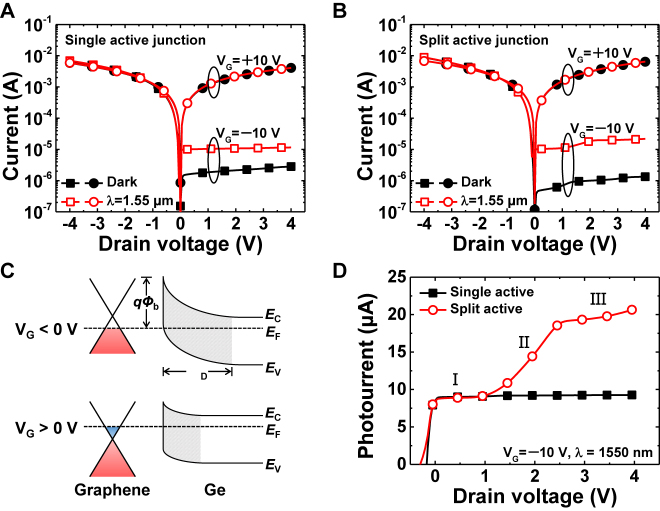
Current–voltage characteristics of the (A) single and (B) split active graphene/Ge Schottky junction photodetectors in the dark and under illumination. (C) Energy band diagrams of the graphene/Ge Schottky junction with gate-modulation at equilibrium (*V*_D_ = 0 V). Here, *Φ*_b_, *E*_C_, *E*_V_, *E*_F_, and *W*_D_ represent the Schottky barrier height, conduction band edge, valence band edge, Fermi level, and depletion width, respectively. (D) Linear photocurrents for the graphene/Ge Schottky junction photodetectors at *V*_G_ = −10 V. Photoresponse characteristics were measured at a wavelength of 1550 nm with an incident light power of 10 μW.

Figure 2A and B shows the *I*–*V* characteristics of the single and split active graphene/Ge Schottky junction photodetectors in the dark and under illumination, respectively. The dark currents of both devices had the characteristics of a typical Schottky junction and were substantially modulated by the gate bias (Figure 2A and B, black lines). In both cases, the rectification ratios increased by more than four orders of magnitude at a drain bias of ±2 V with the top-gate modulation of the Schottky barrier. In addition, the photocurrents, defined as *I*_photo_ = *I*_illumination_ − *I*_dark_, significantly increased at a negative gate bias of −10 V, but were negligible at a positive gate bias of 10 V (Figure 2A and B, red lines). Figure 2C shows the energy band diagrams illustrating the gate modulation mechanism of the Schottky barrier height. The Fermi level of graphene is sensitive to gate bias; thus, the height of the Schottky barrier changes as a function of gate bias. When a negative bias is applied to the gate, the Fermi level of graphene decreases; thereby, increasing the height of the graphene/Ge Schottky barrier. Moreover, the depletion width in the Ge substrate increases, causing additional band bending and an increase in the hole concentration of graphene [15]. In contrast, with a positive gate bias, the Fermi level of graphene shifts upward, and the barrier height decreases; thus, an ohmic-like behavior develops.

Figure 2D shows the photocurrent curves on a linear scale at a negative gate bias of −10 V for single and split active junction photodetectors. The photodetector with a single active region had a nearly constant photocurrent under infrared illumination, regardless of the drain bias. In contrast, the *I*–*V* curve of the photodetector with the split active junction structure had nonlinear characteristics with several linear parts of different slopes, depicted as regions I, II, and III, which correspond to different current-transport mechanisms. Region I corresponds to thermionic emission through the Schottky barrier as the Schottky junction mechanism. In region II, the kink appeared when the applied voltage exceeded 1 V; this region corresponds to the carrier multiplication in Ge owing to the high internal electric field caused by the high drain bias. A high electric field strength could accelerate charge carriers in the depletion region and result in carrier multiplication, such as impact ionization. This effect causes a nonlinear increase in the current as the applied bias increases. In region III, where the drain bias was higher than 2.5 V, the increase in photocurrent was significantly reduced because the effect of carrier multiplication was suppressed. This trend can be attributed to an increase in non-radiative recombination through deep defect centers or Auger recombination [30, 31].

**Figure 3:**
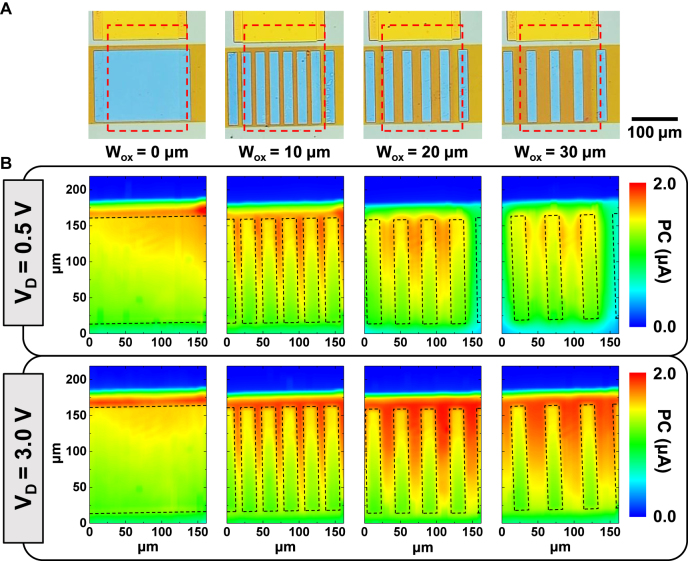
(A) Optical images of the photodetectors with various oxide widths (*W*_ox_ = 0, 10, 20, or 30 μm). Red dashed line: area in which scanning photocurrent microscopy (SPCM) was measured. (B) SPCM images of the scanned area measured by a 1550 nm laser of 10 μW. Photocurrent measured at *V*_D_ = 0.5 V (above) and 3 V (below) under *V*_G_ = −10 V, respectively. Black dashed rectangle: graphene/Ge junctions. The scale of the photocurrents is the same for all measurements.

To further investigate the effect of the split active regions, the spatial distribution of the photocurrents was measured using the SPCM. Figure 3A shows the optical images of the photodetectors with different oxide widths of 0–30 μm. The device was scanned at a size of 3 μm per pixel in a total scanned area of 162 μm  × 219 μm. Figure 3B shows the SPCM images for drain biases of 0.5 and 3 V at a gate bias of −10 V in each device. Regardless of the drain bias, photocurrents were higher in the graphene/SiO_2_/Ge region than in the graphene/Ge region for all devices. In particular, a remarkable photoresponse was observed in the graphene/SiO_2_/Ge regions at a drain bias of 3 V. These results suggest that a large photocurrent is generated in the graphene/SiO_2_/Ge regions owing to the self-gating effect of the graphene/SiO_2_/Ge capacitors by the drain bias. These phenomena can be explained by the formation of an inversion layer underneath the graphene/SiO_2_/Ge structure of the devices [20, 21]. In addition, the formation of an inversion layer increases the concentration of minority carriers near the surface, thereby reducing surface recombination at the oxide–semiconductor interface and consequently improving the photocurrent [32].

**Figure 4:**
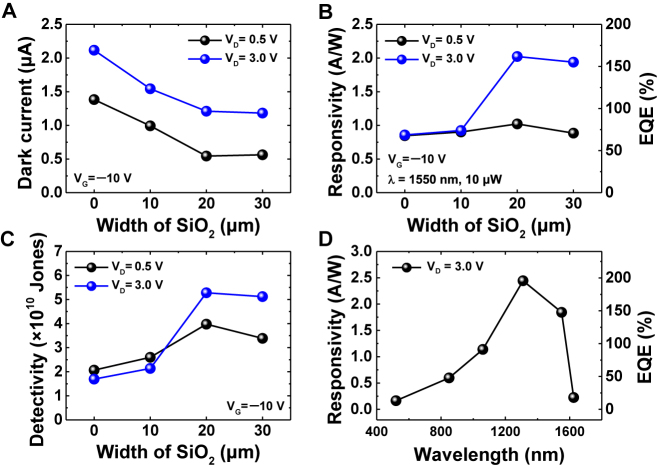
(A–C) Performance comparison of split active junction photodetectors as a function of SiO_2_ width: (A) dark current measured at *V*_D_ = 0.5 and 3 V under *V*_G_ = −10 V; (B) responsivity (left axis) with calculated EQE (right axis); (C) specific detectivity measured at a wavelength of 1550 nm with an incident light power of 10 μW. (D) Wavelength-dependent responsivity (left axis) and EQE (right axis) of the split active junction photodetector measured at a fixed light intensity of 25 μW.

Figure 4A illustrates the dark current trends of split active junction photodetectors with different oxide region widths measured at drain biases of 0.5 and 3.0 V under a gate bias of −10 V. As the oxide width increased, that is, as the area of the graphene/Ge junction decreased, the dark currents were suppressed for both drain biases. This result is consistent with the classical Schottky junction theory, which states that the reverse leakage current caused by the diffusion of minority carriers is proportional to the area of the Schottky junction [33].

To quantitatively analyze the performance of the photodetector, the responsivity *R*, external quantum efficiency EQE, and specific detectivity D^*^ were calculated using the following equations:

(1) $$R=\frac{I_{\text{ph}}}{P_{\text{in}}},\text{ EQE}=R\frac{hc}{q\lambda },\;D^{*}=R\sqrt{\frac{A}{2qI_{\text{dark}}}}\text{,}$$

where *I*_ph_ is the photocurrent, *P*_in_ is the power of the incident light, *h* is Planck’s constant, *c* is the speed of light in a vacuum, *q* is the elementary electric charge, *λ* is the wavelength of the light, *I*_dark_ is the dark current, and *A* is the area of the device. The photosensitive area was calculated to be 160 μm × 165 μm for all devices, including the areas of the graphene/Ge region and the graphene/SiO_2_/Ge region. The performance trends shown in Figure 4B and C suggest that the split active junction photodetector with an oxide width of 20 μm is the most efficiently designed structure, which has the highest responsivity and detectivity. In particular, the responsivity and specific detectivity drastically increased at a drain bias of 3 V, resulting from the formation of the inversion layer in the graphene/SiO_2_/Ge region. As a result, at a gate bias of −10 V and a drain bias of 3 V, a responsivity of 2.02 A W^−1^ with an EQE of 162% and specific detectivity of 5.28 × 10^10^ Jones were achieved. The reduced responsivity in devices with oxide widths greater than 20 μm suggests that the photocarriers generated in the graphene/SiO_2_/Ge regions are farther away from the graphene/Ge junction, resulting in a weaker lateral electric field and consequently difficult to extract into the graphene/Ge junction before carrier recombination. Figure 4D shows the spectral responsivity and EQE of an optimized photodetector in the range of 520–1625 nm at a constant light intensity of 25 μW. The photodetector has peak sensitivity at a wavelength of 1310 nm, which is attributed to the intrinsic light absorption at germanium.

**Figure 5:**
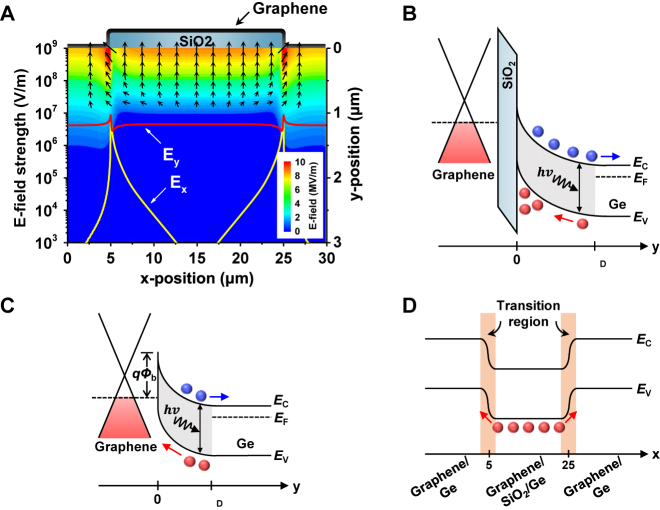
(A) Two-dimensional numerical simulation of the electric field between graphene/Ge and graphene/SiO_2_/Ge region under reverse bias condition (*V*_D_ = 4 V). Line profiles exhibit the *x*- and *y*-components of the electric field (*E*_*x*_ and *E*_*y*_) 10 nm below the Ge surface along the *x*-direction. Black arrow: the electric field strength and direction in the Ge substrate. Schematic energy band diagrams of (B) the edge of the graphene/SiO_2_/Ge region and (C) the middle of the graphene/Ge Schottky junction along the *y*-direction. (D) Lateral band diagram along the Ge surface. Blue and red circle: photogenerated electron and hole in Ge, respectively. Here, *Φ*_b_, *E*_C_, *E*_V_, *E*_F_, and *W*_D_ represent the Schottky barrier height, conduction band edge, valence band edge, Fermi level, and depletion width, respectively.

A numerical simulation of the local electric field was performed to better understand the physical mechanisms of the enhanced photocurrents in the split active junction of the photodetectors. An ideal Schottky junction was modeled in two dimensions under a reverse bias of 4 V, where graphene with a work function of 4.5 eV was placed on the top of a 100-nm-thick SiO_2_ patterned to a width of 20 μm on an *n*-type Ge substrate with a doping concentration of 4.7 × 10^15^ cm^−3^. Figure 5A shows the simulated electric field strength and direction inside the Ge substrate, and the line profiles of the *x*- and *y*-components of the electric field (*E*_*x*_ and *E*_*y*_) along 10 nm below the Ge surface. The electric field was strengthened at the edges of the SiO_2_ region. In particular, *E*_*x*_ was significantly enhanced at the edges of the SiO_2_ region compared to the middle, resulting in an in-plane electric field outside the SiO_2_ region. The direction of this electric field caused the photogenerated carriers to migrate from the edge of the graphene/SiO_2_/Ge region to the graphene/Ge junction. In contrast, the electric field in the middle of the SiO_2_ region was dominated by an almost constant *E*_*y*_, whereas *E*_*x*_ was negligible. This electric field distribution induced a diffusion process rather than a drift process of photocarriers generated in the middle of the graphene/SiO_2_/Ge region; the detailed process will be described later. These simulation results demonstrate that the origin of the enhanced photocurrent is caused by a series of transport processes, in which photogenerated holes in Ge below the SiO_2_ drift and diffuse into the Ge window region. First, the photogenerated holes preferentially travel vertically and accumulate at the SiO_2_/Ge interface by *E*_*y*_. Thereafter, because *E*_*x*_ is weak, the holes diffuse horizontally to the edge of the graphene/SiO_2_/Ge region. Finally, the holes are accelerated by the strong electric field in the transition region between graphene/SiO_2_/Ge and graphene/Ge and collected into the graphene/Ge Schottky region.

The schematic energy band diagrams derived from this understanding are shown in Figure 5B and C as a function of depth from the Ge surface at the edge of the graphene/SiO_2_/Ge region and the middle of the graphene/Ge junction, respectively. Figure 5D shows a lateral energy band diagram along the Ge surface. The accumulated hole layer on the Ge surface caused energy band misalignment between the graphene/SiO_2_/Ge region and the graphene/Ge junction. The photogenerated minority charge carriers were extracted into the graphene channel because of the lateral electric field in the transition region, and carrier multiplication might occur under a strong electric field greater than the breakdown electric field of 10^5^ V cm^−1^ for Ge [34, 35]. The misalignment of the energy band and enhancement of the electric field in the transition region qualitatively indicate that strong energy band bending in Ge significantly affects the transport of photogenerated carriers in the split active junction.

**Figure 6:**
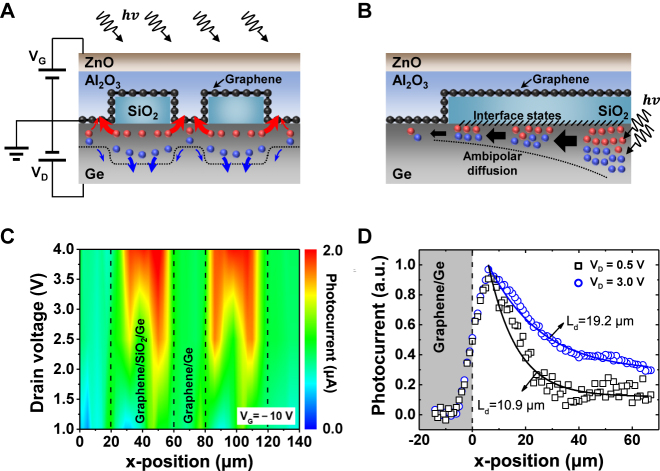
(A) Schematic diagram of the carrier generation, extraction, and collection mechanisms in a split active junction photodetector under illumination. Blue and red circle: photogenerated electron and hole in Ge, respectively. (B) Schematic diagram of the ambipolar diffusion along the SiO_2_/Ge interface. The interface states are presented at the interface between SiO_2_ and Ge. (C) Photocurrent map as a function of *V*_D_ from 1 to 4 V under *V*_G_ = −10 V at incident light power of 10 μW. Black dashed line: transition region. (D) Photocurrent decay profile as a function of the incident light position in the graphene/SiO_2_/Ge region measured at *V*_D_ = 0.5 and 3 V under *V*_G_ = −10 V. Solid lines are a fit to the data, and the diffusion lengths are 10.9 and 19.2 μm at *V*_D_ = 0.5 and 3 V, respectively.

Figure 6A illustrates a scheme that depicts the transport mechanisms of photogenerated carriers, according to the band diagrams derived from the simulation results. Although the photogenerated holes in the graphene/Ge Schottky region were immediately extracted through the graphene channel, the photogenerated holes at the edge of the graphene/SiO_2_/Ge region must travel the distance to the graphene/Ge Schottky junction. In contrast, the transport of photogenerated holes in the middle of the graphene/SiO_2_/Ge region was dominated by ambipolar diffusion. Figure 6B shows the diffusion process in which some of the electron–hole pairs recombine along the SiO_2_/Ge interface. The photocurrent continuously decreased as the photogenerated carriers diffused along the SiO_2_/Ge interface because carrier recombination was increased by the interface states along the interface.

Figure 6C shows the drain bias-dependent photocurrent map at a gate bias of −10 V in the split active junction device with an oxide width of 40 μm. As the drain bias increased, the photocurrent remarkably increased in the graphene/SiO_2_/Ge region, and the photocurrent was greatest at the edge of the graphene/SiO_2_/Ge region. Under a high drain bias, the depletion region widened owing to the increased band bending in the transition region, resulting in more photocarrier generation. As a result, the photogenerated carriers could be extracted more efficiently at the edge of the graphene/SiO_2_/Ge region compared to the middle of the graphene/Ge Schottky junction.

A detailed analysis of the position-dependent photocurrent line profile was performed using the SPCM, as shown in Figure 6D. The measured photocurrent *I*_ph_ in the graphene/SiO_2_/Ge region as a function of position *x* is described as the number of excess carriers collected by the graphene channels. The diffusion length of the photogenerated carriers was calculated directly from the photocurrent decay for different drain biases of 1 and 3 V. In the absence of an external electric field, the collected photocurrent analytically leads to a simple one-dimensional exponential function for *x* > 0 as follows [36, 37]:

(2) $$I_{\text{ph}}=\frac{qG}{2}\text{exp}\left(-\frac{x}{L_{\text{d}}}\right)\text{,}$$

where *I*_ph_ is the photocurrent, *q* is the elementary electric charge, *G* is the carrier generation rate, and *L*_d_ = (*D*_p_*τ*_p_)^1/2^ is the diffusion length with the diffusion coefficient of hole *D*_p_ and the lifetime of hole *τ*_p_. The decay profile of the photocurrent followed a simple exponential function with diffusion lengths *L*_d_ = 10.9 and 19.2 μm at drain biases of 1 and 3 V, respectively. The diffusion length increased at higher drain biases, which was consistent with the expectation that the strong band bending in the transition region affected the transport of photogenerated carriers. This correlation suggests that the split active junction structure should be designed with appropriate dimensions in consideration of the internal electric field according to the thickness of the oxide or the operating voltage to optimize the photodetector.

## 4 Conclusions

The responsivity of the graphene/Ge photodetector increased with a reduced dark current by splitting the active region into a graphene/SiO_2_/Ge structure to increase the EQE. These changes increased the EQE to 162%, the responsivity to 2.02 A W^−1^, and the specific detectivity to 5.28 × 10^10^ Jones. A simulation study indicated that a strong internal electric field was formed beneath the graphene/SiO_2_/Ge regions and helped to generate and efficiently collect photogenerated carriers. Advances in the structural understanding of these devices without additional processes provide a promising approach for future high-performance optoelectronics.

## Supplementary Material

Supplementary Material
