# Supplementary material for: High-performance near-infrared photodetectors based on gate-controlled graphene–germanium Schottky junction with split active junction
Source: Nanophotonics. 2022 Jan 7;11(5):1041–9. doi: 10.1515/nanoph-2021-0738 (PMC11501318; doi:10.1515/nanoph-2021-0738)
Supplement: Supplementary file 1 — Supplementary Material [file j_nanoph-2021-0738_suppl.docx]

Supporting Information

**High-Performance Near-Infrared Photodetectors based on Gate-Controlled Graphene–Germanium Schottky Junction with Split Active Junction**

*Cihyun Kim, Tae Jin Yoo, Min Gyu Kwon, Kyoung Eun Chang, Hyeon Jun Hwang, and Byoung Hun Lee**

1. **Circular transmission line method**

Circular transmission line method (CTLM) test structures were fabricated to determine the quality of the contacts. A CTLM structure comprises of an inner circular contact surrounded by an annular gap within a larger contact area. Each measurement set contained multiple CTLMs with varying gap sizes. The measured total resistance can be expressed as

$R_{\mathrm{tot}}=\frac{R_{\mathrm{sh}}}{2\pi}\left( \ln\left( \frac{L+d}{L} \right)+\frac{L_{T}I_{0}\left( L/L_{T} \right)}{LI_{0}\left( L/L_{T} \right)}+\frac{L_{T}K_{0}\left( \left( L+d \right)/L_{T} \right)}{\left( L+d \right)K_{1}\left( \left( L+d \right)/L_{T} \right)} \right)$, (S1)

where *I*_0_, *I*_1_, *K*_0_, and *K*_1_ are the modified Bessel functions, *L*_T_ is the transfer length, *L* is the radius, and *d* is the gap spacing. When$L>4L_{T}$, then $\frac{I_{0}\left( L/L_{T} \right)}{I_{0}\left( L/L_{T} \right)}$ and $\frac{K_{0}\left( \left( L+d \right)/L_{T} \right)}{K_{1}\left( \left( L+d \right)/L_{T} \right)}$ can be approximated as unity; therefore, Equation S1 simplifies to:

$R_{\mathrm{tot}}=\frac{R_{\mathrm{sh}}}{2\pi}\left( d+2L_{T} \right)C$, $C=\frac{L}{d}\ln\left( 1+\frac{d}{L} \right)$, (S2)

where *C* is the correction factor.

For each contact-resistivity measurement, the resistances of multiple CTLM structures with different gap spacings *d* were measured. *R*_tot_ is a nonlinear function of *d*; however, *R*_tot_/*C* yields a linear function of *d* with a slope of *R*_sh_/(2π) with $d=-2L_{T}$ when *R*_tot_/*C* = 0. The sheet resistance and transfer length can then be extracted from a linear fit to *R*_tot_/*C*.

Figure S1 shows the measured total resistance as a function of the gap spacing and corrected resistance *R*_tot_/*C* with a linear fit to the corrected data. From the linear fit, the values of *L*_T_ and *R*_sh_ can be extracted, and the contact resistivity *ρ*_c_ can be calculated as

$\rho_{c}=L_{T}^{2}\times R_{\mathrm{sh}}$ (S3)


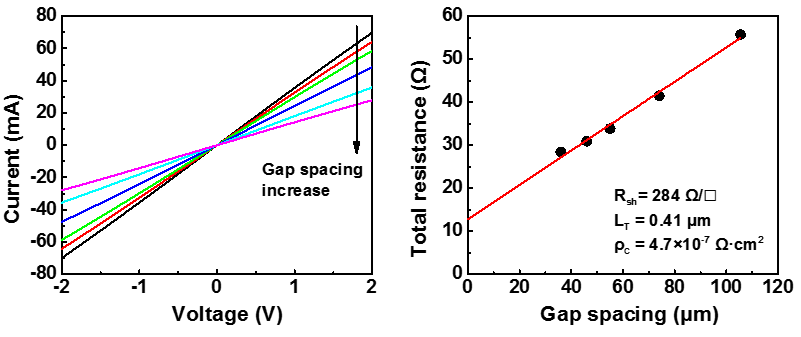


**Figure S1**. Resistance values for a CTLM structure with the linear fit are used to calculate the contact resistivity and sheet resistance. In this study, a 45 nm AgSb alloy (Ag with 1% Sb) was formed on the Ge region using a lift-off process, followed by the rapid thermal annealing process for 5 min in a N_2_ atmosphere at 450 °C.

1. **Setup for measuring the photocurrent characteristics**

A continuous-wave laser beam with a wavelength of 1550 nm from the solid-state laser diode and controller (Thorlabs LPSC-1550-FC and CLD1010LP) was focused onto the sample through a 40× objective lens (Olympus LUCPlanFLN, NA=0.6). The electrical measurements were performed using a Keithley Model 4200 parameter analyzer. The incident light power was 10 μW. A Newport Model 1918-C hand-held optical power meter and a 919P-003-10 thermopile sensor were used to measure the optical power. All measurements were performed under ambient conditions (*T* = 300 K, *P* = 1 atm) in air.


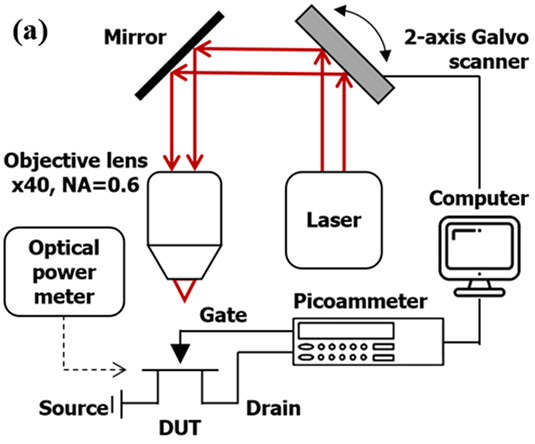

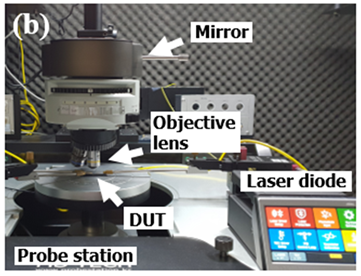


**Figure S2**. Diagram of the photocurrent measurement setup.
